# Comparison of venous morphology, chronic venous disease stage, and venous symptoms in high-impact activities versus low-impact activities

**DOI:** 10.1016/j.jvsv.2026.102559

**Published:** 2026-06-23

**Authors:** Samuel Béliard, Laurent Mourot, Hélène Thomas, Nicolas Tordi, David Ferreira

**Affiliations:** a Cabinet Cardioptim - Clinique Saint Vincent, Groupe Elsan, Besançon, France; b Université Marie et Louis Pasteur, SINERGIES, Besançon, France; c Université Marie et Louis Pasteur, Plateforme EPSI, Besançon, France; d Department of Biological Sciences, Thompson Rivers University, Kamloops, BC, Canada; e Centre Hospitalier Louis Pasteur, Dole, France; f Université Marie et Louis Pasteur, EFS, INSERM, UMR RIGHT, Besançon, France; g Département d'Anesthésie Réanimation Chirurgicale, Université Marie et Louis Pasteur, INSERM, UMR 1322 LINC, CHU de Besançon, Besançon, France

**Keywords:** Physical activity, Symptoms and signs, Ultrasonography, Varicose veins

## Abstract

**Background:**

Regular physical activity is widely recommended to improve venous return and prevent chronic venous disease. However, some guidelines and empirical recommendations distinguish between low-impact (LI) activities, considered beneficial, and high-impact (HI) activities, sometimes discouraged because of their presumed harmful effects on venous valves. These warnings are largely based on historical observations rather than robust scientific evidence. The objective of this study was to evaluate whether HI activities are associated with a higher prevalence of varicose veins compared with LI activities in individuals complying with World Health Organization physical activity recommendations.

**Methods:**

This cross-sectional analysis was conducted within the VARISPORT cohort. Adults aged 18 to 80 years who reported at least 150 minutes of physical activity per week for a minimum of 6 months were included. Participants were classified into LI activities (cycling, swimming) or HI activities (running, team sports) groups according to their main activity. All participants underwent standardized clinical and Doppler ultrasound examination. The primary outcome was the prevalence of varicose veins, defined as superficial veins >3 mm with pathological reflux. Secondary outcomes included venous symptoms (Carpentier score); Clinical, Etiology, Anatomy, and Pathophysiology classification; and diameters of deep and superficial lower-limb veins.

**Results:**

A total of 161 participants were included (53 LI activities, 108 HI activities). The presence of varicose veins did not significantly differ between the two groups, eight participants (15%) in the LI group and 17 participants (16%) in the HI group (odds ratio, 1.05; 95% confidence interval, 0.39-3.0; *P* = .99). No significant differences were observed regarding symptoms; Clinical, Etiology, Anatomy, and Pathophysiology stage class distribution; or venous calibers. In multivariate analysis, age over 60 years, family history of varicose veins, and high weekly training volume were independent risk factors, whereas the HI or LI nature of activity was not.

**Conclusions:**

In individuals meeting World Health Organization physical activity guidelines, HI activities are not associated with a higher prevalence of varicose veins compared with LI activities. These results challenge longstanding recommendations discouraging certain HI exercises and support the promotion of regular physical activity, regardless of its impact level, for the maintenance of venous health.

**Clinical Relevance:**

In this cross-sectional study of 161 physically active adults meeting the World Health Organization recommendations, no significant association was observed between weight-bearing and non-weight-bearing physical activities and the prevalence of varicose veins; venous symptoms; Clinical, Etiology, Anatomy, and Pathophysiology stage; or lower-limb venous diameters assessed by duplex ultrasound.

**Article Highlights**

• **Type of Research:** Single-center, retrospective cross-sectional cohort study.
• **Key Findings:** The presence of varicose veins did not significantly differ between the two groups, eight participants (15 %) in the low-impact activities group and 17 participants (16%) in the high-impact activities group (odds ratio, 1.05; 95% confidence interval, 0.39-3.0; *P* = .99).
• **Take Home Message:** No significant association was observed between high-impact activities and low-impact activities and the prevalence of varicose veins; venous symptoms; Clinical, Etiology, Anatomy, and Pathophysiology stage; or lower-limb venous diameters assessed by duplex ultrasound.

Generally speaking, regular physical activity is considered to be beneficial to venous return in the legs.^1^ Thus, practicing a regular physical activity, either leisure or moderate, is recommended for primary, secondary, and tertiary prevention of varicose veins.^2^, ^3^, ^4^ The observational studies by Chatard et al suggest that practicing physical activities is a significant preventive factor against venous stasis and insufficiency and also limits the progression of chronic venous disease (CVD).^5^^,^^6^

The most favorable physical activities in these cases are repetitive motion physical activities requiring dynamic and rhythmic effort.^7^^,^^8^ Thus, walking on soft surfaces, hiking, gentle workouts, biking, swimming, and cross-country skiing are considered to be beneficial.^9^ The goal of these recommendations is mainly to improve venous hemodynamics and reduce distal venous hypertension by activating the plantar and calf venous pumps as well as to fight against weight gain and a sedentary lifestyle.^8^^,^^10^ During supervised physical activities, venous compliance was found to improve,^11^^,^^12^ and endothelial function was optimized.^13^ Also, one recent study showed that athletes practicing more than 6 hours of endurance physical activities per week had physiological adaptations that increased their venous capacitance and improved venous drainage mechanisms.^14^ This is the notion of athletes' veins,^14^^,^^15^ similar to the adaptations that have been shown in the rest of the cardiovascular system, with the athletes' heart^16^ and athletes' arteries.^17^

The World Health Organization (WHO) recommends for adults: at least 150 to 300 minutes of moderate-intensity aerobic activity or at least 75 to 150 minutes of intense aerobic physical activity or an equivalent combination of moderate and intense physical activity per week.^18^ The goal is clearly to limit inactivity and to carry out primary and secondary prevention of cardiovascular diseases.^19^

Since the 1980s, some physicians recommend so-called low-impact (LI) activities, such as biking or swimming, to limit sudden increases in pressure in the veins of the lower leg (LL), and on the other hand, do not recommend so-called high-impact (HI) physical activities, such as running, tennis, or collective physical activities, that accumulate stress on the LL veins because of the repetitive impact of each step or jump.^20^^,^^21^ In this study, we define “HI activities” as physical activities involving repetitive ground impact forces transmitted through the lower limbs (eg, running, team sports, tennis) and “LI activities” as those without significant ground impact (eg, cycling, swimming).

Such practices are still recommended, as the French National Health Insurance advises against certain physical activities in patients with varicose veins^22^ in particular:

• HI physical activities that are performed on hard surfaces that alter the valvomuscular mechanism by creating sudden increases in venous pressure and cause microtraumas (marathons, tennis, basketball, etc).
• Physical activities that block the circulation because of the athlete's posture, clothing, or efforts with a closed glottis; for example, horseback riding, judo, downhill skiing, weightlifting, or hockey.

Similarly, the U.K. Vein Clinic^23^ informs its patients that the stress that some sports put on the legs can indeed increase the risk of forming varicose veins. Some of the more stressful sports include distance running (long periods of remaining upright can cause blood to pool in the LL), skiing, and snowboarding (intra-abdominal pressure can damage vein valves), tennis, basketball, netball (any sport that includes rapid stop-start motions with the legs can damage vein valves), football (physical contact between players can damage vein valves or break and worsen existing varicose veins), and weightlifting (strain of lifting weights can damage or worsen already damaged veins).

A careful review of these earlier data^24^ indicates that they were largely based on observational methods, such as staged tourniquet examinations, without the support of Doppler ultrasound assessment. In addition, the authors did not report any functional consequences, as no symptoms were described in the population of athletes studied.^25^ To date, the hypothesis that repeated impacts or jolts on hard surfaces during HI physical activity may cause structural damage to venous valves has never been scientifically demonstrated. As a result, these longstanding recommendations, which rely on historical empirical assumptions, may be misleading and could inadvertently discourage individuals from engaging in physical activity, particularly those who do not favor activities, such as cycling or swimming.

Consequently, to help clinicians provide the best possible advice to people who want to engage in regular physical activity, the objective of the present study was to evaluate whether HI physical activities are associated with a higher prevalence of varicose veins compared with LI physical activities in participants who comply with WHO recommendations for physical activity.

## Materials and methods

### Background

The VARISPORT study was performed at the Dole Hospital and the Exercise Performance Health Innovation platform from 2015 to 2020. The study was reported in accordance with the Strengthening the Reporting of Observational Studies in Epidemiology rules.^26^ It was approved by the Local Ethics Committee (CPP Est III, registration number 2015-A00526-43) and registered in the Clinical Trials (NCT02846051). Written and oral informed consent was obtained from all participants before they were included.

### Conception of the study

In this nested design derived from the VARISPORT cohort, we conducted a cross-sectional comparison of lower-limb venous status between individuals practicing LI physical activities and those engaged in HI physical activities. The original VARISPORT sample size was based on a presumed 40% prevalence of varicose veins in the general population and a 20% higher prevalence in highly trained individuals (more than 8 hours weekly), requiring 120 participants per group. High physical activity volume group participants had been matched to controls on gender, age, and body mass index (BMI).

For the present exploratory analysis, only participants reporting more than 150 minutes of weekly physical activity, as recommended by the WHO,^18^ were included. The primary outcome was the prevalence of varicose veins (>3 mm superficial veins with significant reflux).^27^ Secondary outcomes included symptoms (Carpentier score), clinical status (Clinical, Etiology, Anatomy, and Pathophysiology [CEAP] classification), and venous morphology (diameter of deep and superficial lower-limb veins) in both activity groups.

### Inclusion and exclusion criteria

Participants included in the study were volunteers aged 18 to 80 years old. They were divided into two groups (*LI and HI groups*) according to the following criteria:

• Each participant was questioned about the amount and intensity of his/her physical activity.
• All participants had performed at least >150 minutes of physical activity/wk, with an intensity above the ventilatory threshold, either 60% to 70% of the maximum oxygen concentration rate, 70% to 80% of the maximum oxygen concentration rate, or 70% to 80% of the maximum heart rate.
• The participants in the HI group mainly practiced a physical activity, such as running, football, or handball, whereas the LI group mainly practiced LI physical activities (biking, swimming).

### Data collection

Each participant included in this study underwent a phlebology examination (clinical evaluation and a vascular Doppler ultrasound), performed by the same experienced vascular medicine specialist (S.B.). Participants were questioned about their medical history and venous risk factors (gender, age, tobacco use, and family medical history). More specific venous symptoms of CVD were also looked for using the questionnaire by Carpentier et al^28^ (Table I).

**Table I:** Description of the Carpentier score

Are the symptoms you feel in the legs…	Answer	Points
Similar to a sensation of heavy, weighty, or swollen legs	Yes	1
Associated with impatient legs, itching, or pain in the place of visible veins	Yes	1
Worsened by a hot environment or improved by a cold environment	Yes	1
Worsened by walking	No	1

CVD was categorized for both legs in each participant using the CEAP classification.^29^ Measurements of the caliber (anteroposterior diameter [APD]) and detection of reflux in LL veins were performed with a 7.5 MHz multifrequency linear transducer (L12-3; Philips) connected to a high-resolution ultrasound machine (Affiniti 70; Philips), according to guidelines.^30^ The participants rested for 10 minutes before the examination. Deep LL veins were examined in two standardized measurement zones: the popliteal vein (POP V) and a medial gastrocnemius vein (MG V), when this was the most dilated. The superficial LL veins were examined in two standardized measurement zones: the great saphenous vein (GSV) at the middle third of the thigh, and the small saphenous vein (SSV) at the middle third of the leg. A complete ultrasound examination of the general condition of the LL veins and the extent of any identified reflux was not performed. The larger nonsaphenous vein (NSV) was only assessed when the vascular physician detected an NSV with an APD >3 mm, including tributaries of the saphenous veins as well as superficial veins independent of the saphenous system. The examining sonographer performed manual rapid decompression by using the hand that was not involved in scanning. Compression was performed at the midcalf level to obtain a visible flow velocity increase, followed by rapid release of pressure to the calf.^31^ The duration of reversed flow after a provoking maneuver (reflux time) was measured in the longitudinal view of the vein using a spectral pulse wave Doppler probe angled at 60 (abnormal venous reflux: >0.5 seconds in the superficial veins and >1 second in the deep veins).^32^

### Statistical analysis

The statistical unit chosen for the description of the sample was the participant. The statistical unit chosen for the prevalence of varicose veins, the study of symptoms (Carpentier score), the clinical criteria (CEAP classification), and morphological criteria (vein caliber) was the most severely affected leg.

The results were expressed as means ± standard deviation or medians (interquartile) for quantitative variables according to the case and as frequencies and percentages for qualitative variables. Categorical variables were compared using the *χ*^2^ or Fisher exact test. Normality of quantitative variables was assessed using the Shapiro-Wilk test. For normally distributed variables, group comparisons were performed using Welch's *t*-test, which does not assume equal variances. For non-normally distributed variables, the Kruskal-Wallis test was applied. Logistic regression was performed to determine whether the HI/LI aspect of a physical activity was associated with varices while taking into account confounding factors (quantity of physical activity, gender, BMI, age, and family history of varices) and the collinearity of the variables (inflation factor of variance <2). *P* < .05 was considered significant. All analyses were performed with R software (version 4.5.1).^33^

## Results

One hundred sixty-one participants were included in this study (53 in the LI group and 108 in the HI group). The LI group included 37 participants (70%) who practiced biking, 8 (15%) swimming, 5 (9%) the triathlon, and the 3 (6%) remaining participants practiced horseback riding or canoeing. The HI group included 60 runners (56%) (on the road or trail), 13 (12%) people practicing Nordic walking or hiking, 11 (10%) a team sport (football, handball, rugby, and volleyball), 10 (9%) people practicing CrossFit or weightlifting, and 14 (13%) people participating in cross-country skiing, dancing, or a racket sport. The description of the population is presented in Table II.

**Table II:** Description of the sociodemographic characteristics of the participants in the study

	LI group (n = 53)	HI group (n = 108)	*P* value
Physical activity volume			
2.5-8 h/wk	21 (40)	60 (56)	.07
>8 h/wk	32 (60)	48 (44)	
Gender (women)	10 (19)	41 (38)	.38
Age			
<30	24 (45)	36 (33)	.004
30-60	18 (34)	64 (59)	
>60	11 (21)	8 (7)	
BMI			
<18.5	1 (2)	8 (7)	.41
18.5-25	44 (83)	83 (77)	
>25	8 (15)	17 (16)	
Active smoking	2 (4)	9 (8)	.34
Smoking cessation	8 (15)	19 (18)	.82
History of pregnancy	4 (8)	27 (25)	.16
Previous history of venous thromboembolic disease	1 (2)	6 (6)	.43
Family history of CVD			
Maternal side	14 (26)	37 (34)	.91
Paternal side	6 (11)	14 (13)	.32
Both sides	18 (34)	45 (41)	.39

*BMI*, Body mass index; *CVD*, chronic venous disease; *HI*, high-impact; *LI*, low-impact.

Data are presented as *n* (%).

The presence of varicose veins did not significantly differ between the two groups: eight participants (15%) in the LI group and 17 participants (16%) in the HI group (odds ratio [OR], 1.05; 95% confidence interval, 0.39-3.0; *P* = .99).

There was no significant difference in the distribution of the results of the Carpentier score (Table III) between the two groups (*P* = .89). Based on the evaluation grid proposed for the Carpentier score, 12 of 53 participants or 23% had a score >3 (highly predictive of CVD) in the LI group and 22 of 108 participants or 20% in the HI group (OR, 0.87; 95% confidence interval, 0.37-2.14; *P* = .87).

**Table III:** Carpentier score and Clinical, Etiology, Anatomy, and Pathophysiology (*CEAP*) grades in the LI and HI groups

	LI group (n = 53)	HI group (n = 108)	*P* value
Carpentier score			
0	32 (60)	70 (65)	.89
1	4 (8)	9 (8)	
2	5 (9)	7 (7)	
3	7 (13)	15 (14)	
4	5 (9)	7 (7)	
CEAP grades			
0	0	32 (60)	.37
1	0	7 (13)	
2	0	14 (26)	

*HI*, High-impact; *LI*, low-impact.

Data are presented as *n* (%).

The clinical evaluation of the different stages of CVD with the CEAP scale (Table III) shows that none of the participants in the LI group or the HI group were classified as C3-C4-C5-C6 in this study. The distribution of the three classes, C0, C1, and C2, was not significantly different between the LI and HI groups (*P* = .37).

Table IV presents the calibers (APD, mm) of the veins in the superficial venous networks (GSV, SSV, and larger NSV) and the deep venous network (POP V, MG V).

**Table IV:** Comparisons of lower limb vein diameter (measured as anteroposterior diameter [*APD*]) and the presence or absence of reflux in the low-impact (LI) group vs the high-impact (HI) group

	LI group (n = 53)	WB group (n = 108)	*P* value
APD, mm			
Deep veins			
POP V	11.6 ± 1.5	11.2 ± 1.9	.14
MG V	6.7 ± 1.7	6.3 ± 1.7	.24
Superficial veins			
SSV	3.5 ± 1	3.2 ± 2	.26
GSV	4.3 ± 1	4.3 ± 1.3	.94
NSV	4.6 ± 1.3	5.5 ± 3	.27
Presence or absence of reflux			
Deep veins			
POP V, n (%)	0	0	1
MG V, n (%)	0	0	1
Superficial veins			
SSV, n (%)	4 (8)	2 (2)	.23
GSV, n (%)	6 (11)	11 (10)	.88
NSV, n (%)	14 (26)	18 (17)	.21

*GSV*, Great saphenous vein; *MG V*, medial gastrocnemius vein; *NSV*, nonsaphenous vein; *POP V*, popliteal vein; *SSV*, small saphenous vein.

Data are presented as n (%) or mean ± standard deviation.SS

There was no significant difference between the two groups for the superficial venous network GSV (*P* = .94), NSV (*P* = .27), and SSV (*P* = .26) or for the veins in the deep venous network POP V (*P* = .14) and MG V (*P* = .24).

In the adjusted logistic regression model, high weekly physical activity volume (>8 hours/wk) and family history of CVD were independently associated with the presence of varicose veins (OR >1; *P* = .007 and *P* = .002, respectively), whereas physical activity type (HI vs LI), sex, and BMI showed no significant association (Fig).

**Fig:**
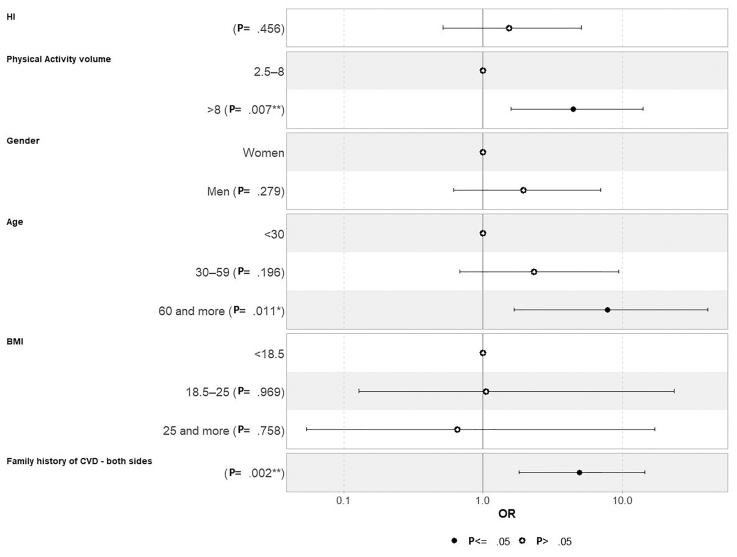
Adjusted odds ratios for varicose veins by patient characteristics and activity level. Multivariable logistic regression analysis showing associations between varicose veins and high-impact (*HI*) activity, weekly physical activity volume, sex, age, body mass index (*BMI*), and family history of chronic venous disease (*CVD*). *OR*, Odds ratio.

## Discussion

To our knowledge, this is the first study suggesting that, among adults meeting WHO physical activity recommendations, HI and LI activities may not differ in their association with varicose vein prevalence. Secondary outcomes, including CEAP classification, symptoms, and venous diameters, showed similar patterns. International recommendations^27^^,^^34^ and recent studies on the effects of physical activity on the veins of the lower limbs^14^^,^^15^^,^^35^ do not distinguish between different types of physical activity: HI vs LI activities. In our study, contrary to older studies on the participants,^20^^,^^21^^,^^24^ the primary end point meets current methodological recommendations: phlebological examination combined with Doppler ultrasound (morphological and hemodynamic criteria).^27^^,^^29^ The number of participants included in our study (161 participants) is significant compared with other recent studies focusing on athletes' veins (eg, only 20 participants in the study by Thomas et al^14^). The two populations compared, LI vs HI, are not different when considering criteria, such as gender, amount of physical activity, BMI, number of pregnancies for women, or family history of CVD. Only the age distribution differs between the two populations, with more participants under 30 and over 60 in the LI group, whereas there are more participants between 30 and 60 in the HI group. All these methodological points reinforce the absence of a significant difference in the prevalence of varicose veins between the two groups. It is interesting to note that the independent risk factors of varices identified on multivariate analysis in our study correspond to the usual risk factors in the literature: age over 60 years old,^36^ a family history of CVD.^37^^,^^38^

Analysis of the Carpentier score shows two groups of participants, one with 0-1-2 in whom the relationship between symptoms and CVD is considered weak, and a second one with a score ≥3, which is highly predictive of CVD.^28^ By applying this analysis to our two groups, we do not find any difference between the LI and HI groups. The Carpentier score can also be used to identify symptomatic (score = 1-2-3-4) from asymptomatic (score = 0) participants. This type of analysis was used by Carpentier et al^28^ as well as in the VARISPORT study.^39^ Based on this method, there is no significant difference between the LI and HI groups in our study (cf. Table III).

The CEAP classification showed no difference in the distribution of the stages of disease between the two groups in our study, with a clear predominance of participants classified as C0.

There was no significant difference in the caliber of superficial or deep veins between the LI and HI groups, suggesting that hypotheses about the harmful role of certain physical activities are not justified.

These results do not support earlier studies on the participant^20^^,^^21^ or the warnings from the French National Health Insurance.^22^ The notion of damage to venous valves because of repeated impacts/jolts on hard ground during HI physical activity has never been proven. Conversely, numerous studies (4, 12, 31, and 31) highlight the relationship between stimulation of the venous pumps (plantar, calf) and optimization of venous return. In this sense, the results of our study should probably encourage us to question the main warnings against certain physical activities that have been given to the general population. Indeed, a message that advises against certain physical activities for varicose veins could lead to inactivity. Even though several studies have clearly indicated that for all age groups and for both genders, varicose veins were significantly more prevalent in the “no regular exercise” group than in the “regular exercise group.”^5^^,^^40^^,^^41^ Unfortunately, only 36% of the population with CVD complies with WHO recommendations for physical activity.^2^

Regarding the association between high-volume physical activity (>8 hours/wk) and varicose veins observed in our multivariate analysis, this finding likely reflects the concept of “athlete's veins” rather than a pathological process. As described by Thomas et al,^14^ athletes practicing intensive endurance activities develop physiological venous adaptations, including increased venous capacitance and larger venous diameters. These adaptations, while meeting our morphological criteria for varicose veins (>3 mm diameter with reflux >0.5 seconds), may represent functional adaptations rather than true pathological venous insufficiency. This interpretation is supported by the absence of participants classified as C3-C6 on the CEAP scale and the lack of significant difference in symptom scores between groups. The reflux observed may result from physiological valve apposition changes because of vein dilatation, as suggested by Van Rij et al,^35^ rather than structural valve damage. For clinical practice, high-volume athletes may present with dilated veins that should not automatically be considered pathological, and the overall cardiovascular benefits of regular physical activity far outweigh any theoretical venous concerns.

The main limits identified in our study were the following:

This study mainly included young men with a normal BMI, limiting the extrapolation of these results. Nevertheless, there was an important difference between the two populations. Indeed, the number of participants in the two groups is different: 53 in the LI group and 108 in the HI group. The difference in the number of participants in the two groups was due to the type of recruitment in this study. The participants evaluated in this study were from the population of the VARISPORT study,^39^ whose goal was to compare the diameter of the veins in the LL and participants' symptoms by comparing high exercise training volume volunteers (more than 8 hours of uninterrupted vigorous intensity physical activity per week) with a volunteer control group matched for age, gender, and body mass index. The 161 participants in our study were selected from the 238 participants in that study.

In this study, we defined varicose veins as superficial veins with a diameter greater than 3 mm and reflux lasting longer than 0.5 seconds. It needs to be identified whether this reflux is the result of pathological valve damage or simply a physiological consequence of veins enlarging with exercise. Van Rij et al^35^ considered that the latter is more likely, with valve apposition being modified by vein dilatation.

It is important to note that this study mainly explored the associations, but not a causal relationship, between the type of physical activities and the presence of varices. Thus, the ORs obtained, even if they were based on an analysis of transversal case-control data, are still pertinent to identify potential correlations. However, it should be kept in mind that because of the case-control design of the study, these measurements do not represent absolute risks or incidences but just the relative strength of the association between exposure and the disease in the study population. These results should be interpreted as indicators of a possible association, which has to be confirmed in prospective studies. This exploratory approach is useful to help understand the factors associated with varicose veins and develop hypotheses for future testing. Sixty percent of participants in the LI group and 44% in the HI group engage in more than 8 hours of physical activity per week. If a significant effect of the type of physical activity (LI vs HI) were to be demonstrated, it would likely be reinforced by the high volume of physical activity among a large proportion of participants in both groups. Conversely, this large proportion of participants with a high volume of weekly physical activity reinforces the fact that there is no significant difference in the criteria evaluated (varicose veins, caliber, and symptoms) in our study.

Our study was not specifically designed to assess the cumulative long-term effects of HI activities on venous health over decades, nor did we capture historical changes in physical activity patterns. Participants were classified based on their current primary activity, and we did not collect data on previous sports practices or musculoskeletal injuries that may have influenced activity choice. However, our study population included participants aged up to 80 years, with a significant proportion over 60 years old, many of whom had been practicing their respective activities for extended periods. Furthermore, our multivariate analysis identified age >60 years as an independent risk factor for varicose veins, yet the type of physical activity (HI vs LI) remained nonsignificant, suggesting that even with prolonged exposure, no detrimental effect of HI activities was observed.

In addition, the revised Venous Clinical Severity Score (rVCSS) was not collected in our study protocol. However, based on the CEAP classification (all participants C0-C2, none C3-C6) and Carpentier symptom scores, we can reasonably infer that rVCSS scores would have been low in our population, reflecting mild or absent clinical disease. Future studies should include the rVCSS as a validated outcome measure.

Our study did not collect data on the use of compression stockings during physical activities. This represents a limitation and an important direction for future research, including randomized controlled trials comparing venous outcomes in athletes using vs not using compression during HI activities.

Future research directions should include (1) prospective longitudinal cohort studies following athletes over 10 to 20 years to establish true causal relationships between activity type and varicose vein incidence; (2) studies incorporating detailed activity history questionnaires documenting lifetime physical activity patterns; (3) mechanistic studies investigating real-time venous pressure measurements during various activities; (4) multicenter studies with larger and more diverse populations; and (5) evaluation of compression therapy during exercise in high-risk individuals.

## Conclusions

In adults adhering to WHO physical activity guidelines, HI physical activities are not associated with a higher prevalence of varicose veins compared with LI activities. This finding is consistent across CEAP classification, symptom profiles, and venous diameters in both superficial and deep lower-limb veins. These results challenge longstanding recommendations discouraging certain HI exercises and support the promotion of regular physical activity, regardless of its impact level, for the maintenance of venous health.

## Funding

None.

## Disclosures

None.
